# Why do patients take part in research? An updated overview of systematic reviews of psychosocial barriers and facilitators

**DOI:** 10.1186/s13063-025-08850-6

**Published:** 2025-05-27

**Authors:** Peter Knapp, Peter Bower, Amber Lidster, Hugh O’Hare, Laura Ferreira Sol, Su Golder, Chris Keyworth, Adwoa Parker, Rebecca Sheridan

**Affiliations:** 1 Hull York Medical School and the Department of Health Sciences, University of York, York, YO10 5DD UK; 2 Centre for Primary Care, University of Manchester, Manchester, UK; 3 Hull York Medical School, University of York, York, YO10 5DD UK; 4 Sheffield Teaching Hospitals NHS Foundation Trust, Sheffield, UK; 5 Department of Health Sciences, University of York, York, YO10 5DD UK; 6 School of Psychology, University of Leeds, Leeds, UK; 7 Department of Health Sciences, York Trials Unit, University of York, York, YO10 5DD UK

**Keywords:** Research, Participation, Recruitment, Barriers, Facilitators, Consent, Psychosocial, Trials

## Abstract

**Background:**

Efficient, equitable health research depends on understanding why people decide to take part. The aims of this overview were to update the version published in 2020, identifying psychosocial influences on participation and mapping them to recruitment research and psychological theory.

**Methods:**

Searches were undertaken in February 2024. Qualitative, quantitative, and mixed-methods systematic reviews were identified, without language or date limits. Methodological quality was rated using AMSTAR-2, and low-quality reviews were excluded. Barriers and facilitators were identified inductively and mapped to the Theoretical Domains Framework (TDF) and COM-B model, and to empirical recruitment research.

**Results:**

The update included 70 reviews, including 44 new reviews, covering a breadth of populations and settings, and drawing on 1940 primary studies (1428 unique).

We identified 15 facilitators, most commonly: altruism, potential for personal benefit and trust. Incentives and convenient, low-burden research were also facilitators. Another 10 facilitators were new to this update.

There were 16 barriers, most commonly: perceived risk, practical difficulties, and distrust of researchers. Many barriers applied to specific designs, particularly randomised trials. Factors that were barriers or facilitators include the influence of others and information quality.

Barriers and facilitators were coded to the Motivation and Opportunity components of the TDF, particularly knowledge and social influences; only two factors were coded to a Capability. Psychosocial influences and empirical recruitment research had some overlap, but some barriers and facilitators had not been evaluated.

**Conclusions:**

Common barriers and facilitators to research participation were identified, some new to this update, which could be addressed through targeted recruitment strategies to increase the efficiency and generalisability of primary research. Factors affecting participation are not only personal; they are also normative and social. The priorities are to change the ways we recruit to research (perhaps tested in SWATs) and identify barriers and facilitators in areas not well covered in current research.

**Trial registration:**

PROSPERO CRD42017062738. Registered on April 2017.

**Supplementary Information:**

The online version contains supplementary material available at 10.1186/s13063-025-08850-6.

## Background

Successful health research is crucial to informed healthcare policy and practice. However, except for studies that draw on routinely collected data, research is dependent on individual decisions to take part. The recruitment and retention of patients and the public to health research continue to be challenging [1–3], and it is vital that we understand why people decide to join research [4], not least because the consequences of poor recruitment can be methodological, ethical and financial.

The methodological effects of poor recruitment are primarily studies failing to reach their target size [5], which reduces statistical power and potentially leads to inconclusive or incorrect results. This problem particularly affects intervention trials but not exclusively: other research designs are also adversely affected by a lack of statistical power [6]. Poor recruitment may also affect sample composition and so the study’s external validity, particularly when recruitment or participation decisions are associated with baseline characteristics [7, 8]. The ethical need to understand why people take part in research stems from the researcher’s duty to obtain valid consent, predicated on participants having a realistic understanding of key aspects of the research, including potential personal benefits, their obligations as research participants, and the societal value of research findings [9]. The financial consequences of poor recruitment tend to be overlooked but may be significant, both directly, by the need to extend study recruitment periods, and indirectly, by research waste and the societal costs associated with the production of poor research [10].

Challenges in recruitment and retention led to the development of SWATs (or Studies Within A Trial) [11], which are studies embedded within a host trial that test research processes such as recruitment methods. In the UK, the SWAT evidence on recruitment and retention interventions has been collated by Cochrane [2, 3, 12], offering a structured appraisal of the effectiveness of a range of interventions. Research participation is a behaviour, potentially understandable by the growing body of theoretical and empirical evidence on behaviour change [9, 13–15]. Therefore, having a better understanding of the reasons why people take part in research offers the possibility of making theory-driven recruitment and retention interventions which can be tested using SWATs to provide a better fit between patients’ motivations, the available research opportunities, and the societal need for valid research.

In 2020, we published a ‘review of reviews’ of the psychosocial barriers and facilitators to research participation, which included 26 reviews reporting 429 unique primary studies [16]. However, several important healthcare settings were not covered in the published research at that time, such as primary care and screening, and we were also aware of the recent publication of significant, relevant research. Therefore, our objective was to update our overview of systematic reviews on the factors that influence individual decisions to take part in research and their links to behavioural theory and recruitment interventions. The update would allow us to include evidence published over the past 5 years, not only to provide greater coverage across patient groups and healthcare settings but also to test the robustness of our 2020 findings.

## Methods

The review update was registered on PROSPERO (https://www.crd.york.ac.uk/prospero/display_record.php?ID=CRD42017062738) and has been reported in accordance with PRISMA and PRIOR guidelines [17, 18].

### Data sources and searches

The aim of the searches was to identify systematic reviews that report the psychosocial factors that influence the decisions of patients and the public on participation in health research. The search strategy used in the 2020 publication was revised slightly, in accordance with changes to database terminology, and after confirming that no new indexing terms had been created that were relevant to our search. The search was run in Medline (Ovid) and then adapted for other databases. Searches were limited to systematic reviews, using Database of Abstracts of Reviews of Effects (DARE) search strategies (see Supplementary Materials for database searches).

The following databases were searched on February 27, 2024 (by SG): Medline, Embase, CINAHL Ultimate, Cochrane Database of Systematic Reviews (CDSR), and PsychINFO. PROSPERO was also searched for ongoing reviews. The searches were updated by running two searches: first, from database inception dates to March 2024, and second, from 1 st January 2019 to March 2024. We did not limit the first search by date to more accurately remove duplicates using the existing original Endnote Library. The two searches were then deduplicated (by subtracting one from the other), using EndNote, to leave a set of results specific to the update. The remaining search results were then deduplicated within to remove articles detected by more than one database, with the resultant hits being exported into Covidence for processing. Later in the process, backwards- and forwards-citation searches were undertaken in Google Scholar on studies due for data extraction.

### Inclusion and exclusion criteria

We used the same inclusion criteria as the 2020 publication.

We included systematic reviews using quantitative, qualitative or mixed methods to report findings from primary studies into patient or public psychosocial determinants of participation in health research. The primary studies included in the reviews could be quantitative, qualitative or mixed methods. Our focus was on real research rather than hypothetical scenarios: reviews in this area often have mixed content, and we only included reviews if at least two-thirds of their primary studies involved actual research. We excluded systematic reviews that only reported the demographic characteristics of research participants, and those limited to health care practitioners’ or researchers’ views on the determinants of participation. When a review reported patients’ and professionals’ views, we included it if patients’ views were reported and could be extracted separately. When the subject was research participation in children or others lacking capacity, we included systematic reviews reporting the views of those responsible for participation decisions, such as parents. No limits were applied to geography or language (and we translated potentially relevant reviews not published in English).

### Screening

Initially we screened titles and abstracts, using pre-determined criteria (that is, reporting the views of research participants or potential research participants; answering a defined research question; using stated inclusion criteria; searching at least three indexed databases), which was done independently by two authors (PK; HO). Then potentially relevant full-text articles were independently screened by two authors (PK; AL). Disagreements at either stage were resolved through consensus.

### Quality assessment

Each review was initially assessed independently by two reviewers (from PK; HO; AL; LS) on AMSTAR-2 [19]. Each of its 11 items was scored 1 (met) or 0 (not met), deriving a range of 0–11. As in our 2020 publication, one minor change to recommended scoring was that, for item 5, articles only had to list included studies and not excluded studies (as few reviews reported excluded studies). Reviews were categorised as AMSTAR-2 low (0–3), moderate (4–7) or high quality (8–11), and low-quality reviews (scoring 0–3) were excluded [20].

### Data extraction and analysis

We adapted the data extraction form used successfully in the 2020 publication, which included: review title and aims, study design and analysis, details of included studies, participant details and key findings. The included systematic reviews were the unit of analysis, and so we did not access content from primary studies included in the reviews; consequently, any information not reported in the systematic review (for example, the sample size of included studies) was recorded as ‘not stated’.

Data were extracted by two researchers working independently (from PK; HO; AL; LS) and findings were reconciled by consensus. The review findings on barriers and facilitators were either extracted verbatim from the review text or summarised using categories assigned by review authors. The findings were then categorised in line with those identified in the 2020 publication, inductively coding them as a barrier (something that impeded recruitment) or a facilitator (something that enhanced or encouraged recruitment). When a factor was identified as a barrier in some reviews and a facilitator in others, we coded it as ‘either a barrier or facilitator’. We also extracted the following data for each included review: aim; focus on barriers or facilitators; search dates; primary study publication dates; population; total number of included primary studies and number of relevant primary studies (if different); combined sample size in the primary studies; number of unique studies; primary study methods; health setting; country of origin of included studies.

Extracted categories were then coded [21] according to the COM-B model [22] and its sub-categories, which is the operational model of the Theoretical Domains Framework (TDF) of behaviours [13, 14]. (coding undertaken by PK and CK). The TDF includes 14 domains that influence individual behaviour, which are clustered into three overarching constructs: Capability, Opportunity and Motivation (the COM-B model) [15]. The TDF and COM-B model are used here because research participation is a behaviour. Capability recognises how psychological and physical capabilities influence behaviour, and it includes these TDF constructs: knowledge; skills; memory, attention and decision processes; and behavioural regulation. Opportunity concerns the influence of the social and physical environment on behaviour, including these TDF constructs: social influences; and environmental context and resources. Motivation refers to conscious and unconscious cognitive processes influencing behaviour, which include these TDF constructs: social or professional role and identity; beliefs about capabilities; optimism; beliefs about consequences; reinforcement; intentions; goals; and emotion.

We also coded identified barriers and facilitators against empirical recruitment interventions that could potentially address them (coded by PK, PB, RS and AP), using interventions that had been subject to SWATs and were included in the Cochrane systematic review of recruitment interventions [3, 12]. We coded them by linking the objective of each SWAT intervention, that is, what it was intended to change, to the identified determinants of participation. We organised recruitment interventions by the evidence of effect, using three categories (as defined by the Cochrane authors): those that probably affect recruitment; those shown not to affect recruitment; and those with uncertain effects.

## Results

**Fig. 1:**
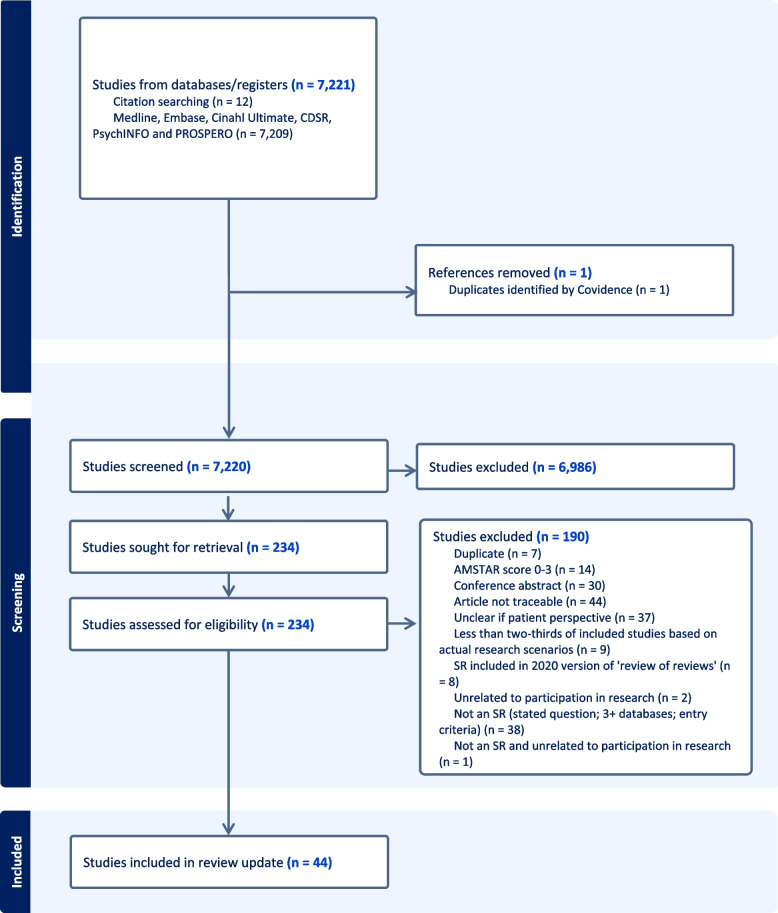
PRISMA flowchart of updated search results

Our searches identified a total of 7208 results, plus another 12 papers added following citation searching (see Fig. 1). We retained 234 articles for full-text review and finally included 44 reviews, which were combined with the 26 review articles included in the 2020 publication to form a dataset of 70 systematic reviews, a 170% increase in total. Details of exclusions are provided in Fig. 1.

### Quality of the evidence

Of the 44 new reviews, 33 were rated as moderate quality on AMSTAR-2 and 11 were of high quality (see Supplementary Materials Table 1). When combined with the previous 26 reviews, this generated 70 AMSTAR-2 scores in the range 4–9, with 17 reviews (24.3%) rated as high quality and 53 (75.7%) rated as moderate. We did not undertake sensitivity analyses according to AMSTAR-2 scores.

In the 2020 publication, the median AMSTAR-2 score of the 26 included reviews was 6.5. Among the 44 new reviews, the median AMSTAR-2 score was 7, suggesting a modest increase in review quality. However, 14 reviews were excluded from this update due to low AMSTAR-2 scores; a big increase from four excluded reviews in 2020. When the excluded reviews were included in the distribution of AMSTAR-2 scores, the median and mean AMSTAR-2 scores in 2020 (from 26 included plus 4 excluded reviews) were 6 and 6.0, respectively. In this update (from 70 included plus 18 excluded reviews), the equivalent AMSTAR-2 scores were 5 and 5.8, respectively. Overall, then, there appears to be a bigger variation in review quality among more recent reviews. Common methodological weaknesses in the 70 reviews were: excluding unpublished research or research not published in English; not accounting for the scientific quality of the primary research when analysing or discussing the findings; not assessing for publication bias; and not reporting the potential for conflict of interest in the review authors and authors of primary studies. Reviews mostly scored well on the other seven AMSTAR-2 items.

### Findings

The 70 reviews [23–92] had been published from 1999 to 2024 and included 1940 primary studies (published 1982–2023), of which 1428 were unique (see Supplementary Materials Table 1).

Overlap across included systematic reviews in an overview of systematic reviews is crucial to consider, given the possibility of primary results being counted more than once, and we assessed for it in several ways. First, we confirmed that no review had its included studies (and, by extension, its own findings) entirely encapsulated within another, larger review. Second, we calculated the Covered Area (CA) and Corrected Covered Area (CCA) statistics, which are recommended as measures of overlap [93], and which were 1.9% and 0.52%, respectively; both values are low, indicating low rates of overlap across reviews. Third, we calculated that 189 (13.2%) of the unique primary studies were included in more than one review (and so 86.8% of them were included in only one review). Finally, for each review we assessed how many of its relevant, included primary studies were unique to that review (see Supplementary Materials Table 1). Levels of uniqueness ranged from seven reviews (10%) that only contained unique studies [29, 45, 59, 65, 76, 83, 90] to 18 reviews (25.7%) in which a minority of included studies were unique [24, 34, 35, 37, 38, 41, 46, 52, 53, 55, 56, 60, 68, 70, 77, 85, 88, 89]; the remaining 45 reviews (64.3%) had a majority of included studies that were unique. Overall, levels of overlap across the 70 systematic reviews do not appear to be problematic.

The 1940 primary studies had been undertaken in 55 different individual countries. Seven hundred forty-one (38.2%) studies were undertaken in the USA, 354 (18.3%) were from the UK, while 92 (4.7%) and 89 (4.6%) were from Canada and Australia, respectively. Thirty-two other countries contributed multiple studies, including the Netherlands (29; 1.5%), New Zealand (21; 1.1%), Sweden (19; 1.0%), South Africa (17; 0.9%), Tanzania (16; 0.9%), Denmark (16; 0.9%), France (12; 0.6%), Italy (11; 0.6%), Thailand (10; 0.5%) and Japan (10; 0.5%). Fifty-seven (2.9%) studies had been undertaken in more than one country, while 319 (16.4%) studies did not report their country of data origin.

A majority of reviews (41; 58.6%) included a mix of qualitative, quantitative and mixed methods primary studies, while 11 (15.7%) included only qualitative studies [32, 41, 50, 52, 56, 69, 72–74, 77, 89], three (4.3%) included only quantitative studies [23, 29, 84], and 15 (21.4%) did not state the design of included studies [31, 35, 36, 44, 48, 49, 55, 65, 68, 78, 81, 83, 85, 87, 91]. Most reviews (58; 83%) reported both barriers and facilitators, while seven (10%) reviews reported only barriers [23, 35, 51, 70, 81, 85, 91], and four (5.7%) reviews reported only facilitators [26, 36, 54, 79]. We included two reviews not published in English: one from China [92] and another from the Netherlands [78].

Settings: 54 (77%) of the reviews were related to specific health conditions or settings: cancer (15 reviews, 21,4%) [24, 37, 38, 42, 43, 46, 51–53, 58, 63, 77, 84, 89, 92], palliative or end-of-life care (three reviews, 4.3%) [26, 31, 32], vaccination (four reviews, 5.7%) [34–36, 69], dementia or memory problems (three reviews, 4.3%) [25, 47, 54], HIV (three reviews, 4.3%) [35, 36, 73] and mental illness (two reviews, 2.8%) [33, 56]. The following settings were covered in one review each: acute care settings [62], smoking cessation [29], musculoskeletal disease [57], cardiovascular disease [66], tropical diseases [83], bronchoscopy [65], chronic wounds [30], Parkinson’s disease [50], rheumatic disease [60], pain [39], epidemics [69], ‘sensitive’ research topics (such as trauma and violence) [76], and primary care settings [71]. Several important healthcare settings that were absent from the 2020 publication have now been included, such as epidemics [69], primary care (aka family practice) [71], men’s health [27] and palliative care [32].

In terms of types of research participants, 13 reviews (18.6%) focussed on child or adolescent participants [23, 27, 33, 38, 41, 42, 45, 53, 58, 75, 76, 86, 90], five (7.1%) focussed on ethnic minorities [40, 47, 48, 59, 82], and two (2.8%) on under-represented groups [43, 59]. Other reviews focussed on specific participant types: four (5.7%) on women only (including three reviews in pregnancy) [49, 64, 66, 87], four (5.7%) on older people [44, 54, 59, 85], and one review each on: research in China [61] or in sub-Saharan Africa [73], carers [28], and men [27].

Forty-two (60%) of the reviews focussed on participation in specific research designs, such as randomised control trials (*n* = 38), including two (2.8%) that focussed on Phase 1 (early phase) trials [37, 88]. Other reviews had a specific focus on longitudinal studies [27], bio-banking [79] and birth cohort studies [49].

The findings of the overview were synthesised narratively, according to the identified barriers and facilitators. The review of reviews identified 15 facilitators of research participation (see Supplementary Materials Table 2), of which three were included in many more reviews than others: altruism, potential for personal benefit, and confidence or trust in the physician or the research. Altruism and potential for personal benefit were identified in a majority of the reviews, and trust was reported in half of them. Convenient or low-burden research, and financial or practical incentives were also identified as factors increasing participation. The other 10 facilitators were new to this review update and were: feeling valued, opportunity for interaction, similarity of staff to patients, sense of duty, personal relevance, promise to disseminate results, raising awareness of condition, spontaneity, trials with a preference arm, feeling nothing to lose. Notable is the predominance in the list of cognitive factors, including trust and feeling valued, and moral factors such as altruism and a duty to participate, which is similar to altruism but distinguished by its focus on obligation rather than the voluntariness which characterises altruism [94]. The potential for personal benefit and incentives are instrumental facilitators.

Six of the 16 barriers to research participation are new to this update. Of the 16 barriers, the three most common were the perceived risk associated with the research, practical difficulties and a distrust of researchers. A significant proportion of barriers were specific to the primary research design being invited to, particularly randomised trial designs, including an aversion to randomisation, uncertainty, treatment preferences, and desire for choice. Other identified barriers were: personal health concerns, unpleasant or emotive procedures, language or cultural barriers, stigma, lack of interest in the research, lack of knowledge about the health condition, feeling coerced, personal experience of abuse or violence, and not feeling like a candidate for research. Aside from trial-specific factors, the identified barriers are a mix of cognitive and practical aspects. There are no identified moral barriers to participation.

The five factors identified as being either barriers or facilitators were the influence of others (such as relatives), the quality of provided information, previous experience of research, attitudes to healthcare, and a sense of hope or futility; the last three of these factors are newly identified in this update.

### Determinants and their links to the theoretical domains framework

All of the barriers and facilitators identified in the 70 reviews link to at least one TDF domain, although there is a clustering on knowledge, social influences, optimism (or pessimism), goals and beliefs about consequences (see Supplementary Materials Table 2). Each of these domains was then mapped to the overarching constructs outlined in the COM-B model. In the case of some barriers or facilitators, two or more elements of COM-B were assigned as it was not apparent that a single theoretical element could explain it.

Amongst the 15 inductively identified facilitators of research participation, 12 of them link to Motivation components of COM-B, predominantly reflective motivations (i.e. conscious decision making) and automatic motivations (i.e. habits or unconscious decisions). Seven of them link to Opportunity components, either social or physical opportunities, while one was linked to a Capability (psychological capability). Amongst the 16 inductively identified barriers, 12 are linked to Motivation facets (mostly reflective motivations but also automatic), with seven linked to Opportunities (mostly social opportunities but also physical opportunities). One barrier was linked to a Capability component (psychological capability). The five factors that could operate either as facilitators or barriers (other people’s influence; information quality; hope or futility; attitude to healthcare; previous research experience) were mapped to Motivation and Opportunity components, mostly reflective motivation and social opportunity.

### Determinants and their links to evaluated recruitment interventions

The 36 identified barriers and facilitators were also linked to recruitment interventions tested within a SWAT, and 11 (out of 15) facilitators were linked to evaluated interventions; all but one of the 16 barriers were linked to recruitment interventions, and all five of the factors that could be barriers or facilitators were linked to interventions (see Supplementary Materials Table 2). As such, only one intervention has been evaluated that could link to altruism (which was identified in 52 systematic reviews), dissemination of results (three reviews), spontaneity (one review), feeling nothing to lose (one review), or personal experience of abuse or violence (one review). While it is hard to conceive of interventions that could link to some of these factors, it should be possible for others to be tested. There are two other notable points to be made. First, many of the recruitment interventions included in the Cochrane review lack sufficient evidence to decide whether they influence recruitment, meaning there is uncertainty about the effectiveness of designing recruitment interventions based on many of the participation influences identified in this review of reviews. Second, there are some recruitment interventions in the Cochrane review [12] that we could not assign to any barriers or facilitators. That is, for some evaluated interventions, there is no clear link to any of the empirically identified influences on participation decisions. Our view is that ‘recruitment science’ needs further empirical support in many areas as well as a clearer rationale for the intended mechanisms of effect of some recruitment interventions.

## Discussion

### Statement of principal findings

The updated review of reviews included 70 systematic reviews (44 new to this update), drawing on a wide range of healthcare settings and participant groups, based on almost 1500 unique primary studies from multiple countries. The review identified 15 facilitators to research participation, of which three were most common (altruism, potential for personal benefit and trust) and 16 barriers, of which the most common were perceived risk, practical difficulties and distrust. Several barriers were specific to participation in trials [55]. Five factors could be either barriers or facilitators. The most common barriers and facilitators identified in the 2020 publication [16] have largely been replicated with the addition of 44 reviews, which in some cases drew on populations and health settings new to this update. However, the addition of 44 reviews has also enabled the identification of new barriers and facilitators.

The identified barriers and facilitators were all linked to elements of the Theoretical Domains Framework and mostly to the Motivation and Opportunity aspects of the COM-B model, and many of them could be linked to evaluated recruitment interventions, although for most interventions the current empirical evidence is far from definitive [12].

### Strengths and weaknesses of the study

The study has several strengths, including its size and rigour. The study has built on the published 2020 version which included 26 systematic reviews, and now includes a large volume of reviews (and their included primary studies), using quantitative, qualitative and mixed methods. The review of reviews followed good practice in systematic reviewing, including multiple database searching, use of entry criteria, and dual, independent decision making and data extraction; furthermore, no language limits were applied. The quality of the reviews was appraised using an established tool [19], and low-quality reviews were excluded.

A possible weakness of the ‘review of reviews’ study design is the overlap in findings that occurs when individual primary studies are included in more than one systematic review [93]. Some of the 1428 primary studies appeared in more than one systematic review, although levels of overlap across reviews were low, and we ensured that no review could be entirely contained within another, larger review. Overlap is a common and almost inevitable occurrence when undertaking a review of reviews and, given that the systematic review is the unit of analysis, there are problems in adjusting the overall findings to account for it. When reviews of reviews are analysed statistically, it may be possible to give a numerical estimate of the effect of overlapping. In the case of narrative reporting, as used here, it is only possible to estimate the level of overlap and acknowledge it.

Excluding low-quality systematic reviews reduced the size of the overall dataset and in this work a total of 18 reviews were excluded. Set against that, we were able to retain 70 reviews and almost 1500 unique primary studies, a huge dataset. Some systematic reviews and ‘reviews of reviews’ opt to deal with quality variation by including all work but downgrading lower-quality studies in the narrative. We took the view that very low-quality work (scoring less than 4 on a 11-point scale) should be excluded, allowing us to retain and draw conclusions only on work with a lower risk of bias, particularly the publication biases associated with excluding non-English language publications and limited searching of databases. Finally, the identified barriers and facilitators of health research recruitment (in general) were linked to interventions tested for their effects on recruitment to trials [12], which is a slight mismatch. However, there is no equivalent systematic review of recruitment interventions to any health research. The study has been strengthened by mapping barriers and facilitators to recruitment interventions that had been subject to evaluation using random allocation, increasing the rigour of the findings. However, recruitment interventions that have not been subject to SWAT evaluation and were not included in the relevant Cochrane review [12] would be missing from this mapping exercise.

### Strengths and weaknesses in relation to other studies, highlighting important differences in results

This is the largest collation of systematic reviews addressing the important topic of the factors that facilitate or impede people’s participation in health research. It represents a substantive increase in the volume of included reviews and primary studies since the 2020 publication that first addressed this question. The key findings of the 2020 publication have been supported [16], with the 70 included systematic reviews identifying similar common barriers and facilitators of participation. However, the number of identified barriers and facilitators has both increased, as has the number of included systematic reviews (by 170%), both of which have given greater clarity to the less common barriers and facilitators, showing which are important, minority themes and which of them occur only very occasionally.

It is notable that almost 40% of the included primary studies were undertaken in the USA; indeed, two-thirds (66.5%) of the 1940 primary studies come from just four countries (USA, UK, Australia and Canada), all of them English-speaking. Our review of reviews included systematic reviews published in a language other than English, but we had no control over the decisions taken by the authors of the reviews themselves: it is likely that some of them excluded non-English language primary studies. Despite the huge number of unique primary studies represented in this work, and the wide range of healthcare settings, the predominance of work from just four countries does question the global application of the findings, a pattern that is also seen in the global distribution of randomised trials [95, 96]. Furthermore, we found only two reviews not published in English, which is surprising given the huge volume of primary research that has been undertaken: it is possible that our searches, which were sensitive and systematic, may have missed some relevant non-English language reviews.

The COM-B model (and the TDF framework) has been used extensively in research [15, 22], and its elements have been mapped here to the identified barriers and facilitators of participation. However, its value can only really be assessed with the evaluation of recruitment (or retention) interventions that are based on behavioural theory, and two recent systematic reviews suggest this has rarely been done, at least not explicitly [12, 97].

### Meaning of the study: possible explanations and implications for clinicians and policymakers

The links between identified barriers and facilitators and empirical recruitment interventions show that some of the identified influences on recruitment have been subject to randomised evaluation; however, many of them remain unevaluated, requiring interest from researchers and funders.

What is notable in the list of barriers and facilitators is the importance of trust and distrust. From their descriptions in included systematic reviews, trust and distrust appear to be distinct, categoric influences on participation decisions, rather than different levels of a single factor. Trusting a researcher, a set of clinicians or a research organisation appears to exert a powerful influence on participation, and distrust appears to be a similarly influential deterrent. This study was not designed to assess the relative strength of identified barriers and facilitators but, from their reporting in some of the included reviews, trust and distrust both appear to be highly influential, consistent with previous accounts of the topic [98–100].

Also notable is the role that other people play in participation decisions, including beliefs about them (their trustworthiness, as above, or their expected behaviour), their influence on decisions (including the roles of healthcare professionals and relatives), and the potential for research participation to bring about social interaction. It seems clear that decisions about research participation are a mix of individualised elements (such as personal beliefs and moral judgements) and elements that depend on or relate to other people and their views [101–103]. The evidence from this study is that research participation decisions are taken by individuals, but the factors that determine them are not only personal: they are also normative and social.

Participant information was found to be both a barrier and facilitator. Information to inform patient consent decisions is tightly regulated and invariably universal [9], and yet patients can have important, individual preferences for information, particularly how much they receive and its complexity [104, 105]. It may be hard to reconcile individual preferences and population-wide provision, although the increasing use of digital media in provision [106] does offer potential for the tailoring or personalisation of information, to improve the validity of consent decisions.

Given those qualifications, there are significant implications of the findings for recruitment to primary research, and the influences of altruism, personal benefit and trust. SWATs have tended to focus on interventions affecting individual decision-making, but that may need to change given the influence of social and normative factors. For example, social and normative influences on participation decisions may mean a role for social media in recruitment, given the way that social media can result in people belonging to (nominal) groups of like-minded people.

The study also has implications for systematic reviews and primary studies in this area. Some elements seem very well researched, including trials and some healthcare settings in the USA and UK, given the number of systematic reviews undertaken and the inclusion of some primary studies in more than one systematic review. However, there continue to be healthcare settings, population groups and countries that remain under-represented in this dataset, and the findings of this work do not necessarily apply there.

### Unanswered questions and future research

The 70 systematic reviews (and almost 1500 primary studies) have covered a very wide range of settings and groups. However, several areas continue to be unresearched to systematic review level. For example, the influences on recruitment to screening or surgical studies remain uncovered and, although research participation rates are much lower in some population groups than in others, the barriers and facilitators of participation of some of those groups (for example, groups not fluent in the majority language, lower income groups, people with learning disability, and groups lacking capacity to give consent) have not been collated in systematic reviews. Reviews have been undertaken on research participation in people from minority ethnic groups [41, 48, 61, 64] although the area remains under-researched given the sustained differences in participation rates between majority and minority population groups and the legacy of historical oppressive and exclusionary research practices in some countries. Set against that point, the updated searches (after a 5-year gap) resulted in a 170% increase in included reviews and a 230% increase in unique primary studies, indicating sustained and growing levels of interest in this topic, although the increases in published work may not be distributed evenly across population groups and healthcare settings.

The COM-B model has been used in this study to try to provide theoretical context for the identified barriers and facilitators of participation. However, it remains unclear whether empirical research using the COM-B model (and the elements of its underpinning framework, the Theoretical Domains Framework) would support the links to stated barriers and facilitators in the ways that we have suggested [15, 107], although two recent studies suggest that the TDF can be used to inform recruitment interventions [108, 109]. Similarly, we have made links between empirical recruitment interventions and the barriers and facilitators of research participation, to better identify those for which there is a need for empirical evaluation.

Some of the included systematic reviews contained a mix of real and hypothetical studies, and we also excluded some reviews because the proportion of hypothetical studies was too high. Given the volume of published primary studies using actual research, the need for further hypothetical studies appears questionable. However, in general there is a continued need for qualitative research into barriers and facilitators, both in terms of primary studies and systematic reviews [55, 110], although the perspectives of research recruiters have been collated in qualitative systematic review [111].

It would also be useful to evaluate the perspectives of others involved in research (particularly healthcare practitioners and researchers) on patient motivations to participate, although these were not the subject of this review, and then compare them to the patients’ perspectives that have been so heavily researched. Practitioners and researchers are not only in conversation with patients before participation decisions are taken but may well be involved in designing studies (including the information used to inform consent), based on a set of assumptions about patients’ motivations for taking part.

## Conclusions

Thirty-six psychosocial reasons for participation in health research were identified, with three main factors identified as facilitators and three main factors identified as barriers. These were drawn from 70 systematic reviews reporting almost 1500 unique primary studies.

All the identified barriers and facilitators could be mapped to psychological theory, and some factors could be linked to empirical evaluations of recruitment interventions, although many factors remain unevaluated.

It is notable that research participation decisions made by patients are not just individual, but also normative and social. Furthermore, participation decisions draw on a range of cognitive, emotional, instrumental and moral influences.

This review of reviews has built on the original 2020 publication of the work [16] in identifying the common influences on research participation decisions and in encouraging the use of ‘recruitment science’ and psychological theory when designing studies. However, some population groups and some healthcare settings remain under-researched, and most of the published research has originated in a small number of countries; both of these findings offer opportunities for future researchers.

## Supplementary Information

Supplementary Materials Table 1. Details of included systematic reviews.

Supplementary Materials Table 2. Barriers and facilitators identified in systematic reviews, mapped to the Theoretical Domains Framework and recruitment interventions.

## Data Availability

Data collected from included systematic reviews can be provided on request.
