# Supplementary material for: Why do patients take part in research? An updated overview of systematic reviews of psychosocial barriers and facilitators
Source: Trials. 2025 May 27;26:174. doi: 10.1186/s13063-025-08850-6 (PMC12107899; doi:10.1186/s13063-025-08850-6)
Supplement: Supplementary file 1 — Supplementary Materials Table 1. Details of included systematic reviews. [file 13063_2025_8850_MOESM1_ESM.docx]

**Supplementary Materials Table 1. Details of included systematic reviews.**

| **Author, year (includes * if new to this update)** | **Review aim (as quoted). Review focus: barriers, facilitators or both.** | **Publication dates of included studies** | **Population** | **Number of included studies (number in full review if different).**  **Sample size.**  **Number of unique included studies** | **Included study designs: quantitative, qualitative or mixed methods studies.** | **Subject of research participation** | **Location of included primary studies** | **AMSTAR score and category** |
| --- | --- | --- | --- | --- | --- | --- | --- | --- |
| Beasant, 2019 (23) * | “To describe the impact of preference on trial recruitment.”  Barriers only. | 1984-2014. | Children (and parents of children) being asked to take part in trials | 52    *N = Not reported*.  Unique studies 39 of 52. | 52 quantitative. | Children's trials | Not stated. | 7; medium |
| Bell, 2015 (24) * | “To summarize the factors and contexts that influence cancer patient decision making related to clinical trial participation.”  Barriers and facilitators. | 1991 – 2012. | Cancer patients | 34 (out of 36)  *N = 34,392.*  Unique studies 12 of 34. | 20 quantitative; 14 mixed methods. | Trials involving experimental cancer drugs (except for one study, which examined patient preferences for a surgical cancer trial) | USA 18; UK 4; Canada 4; Australia 3; Italy, Korea, Taiwan, China, France, all 1 each. | 4; medium |
| Bethell, 2018 (25) * | "To summarize the reported barriers and enablers of engagement of persons with dementia and care partners in research.”  Barriers and facilitators.  " | 1996 -2018. | People with dementia. | 54 papers (reporting 50 studies).  *N = Not reported.*  Unique studies 48 of 54. | 17 qualitative; 30 quantitative; 3 mixed methods. | Dementia. | UK 30; Canada 9; USA 5; Australia 4; New Zealand 1; More than one country 1. | 5; medium |
| Bloomer, 2018 (26) * | “To understand what are dying persons’ perspectives on, or experiences of, participating in research?”  Facilitators only. | 2003 -2013. | Adults who were dying, had a terminal illness, had a short prognosis or were receiving palliative care. | 8 (out of 10). 1 study (Williams) was hypothetical. 1 study (Perkins) did not research barriers and facilitators.  *N = 1,659.*  Unique studies 7 of 8. | 5 qualitative; 3 quantitative. | Dying patients. | Australia 2; USA 2; UK 2; New Zealand 1; Hong Kong 1. | 7; medium |
| Borg, 2024 (27) * | “To identify specific barriers and facilitators of male recruitment and retention as part of longitudinal research-related studies.”  Barriers and facilitators. | 1976 -2023. | Males aged 17-59, including those consenting on behalf of their children. | 24  N = Not reported.  Unique studies 23 of 24. | 22 quantitative; 2 mixed methods. | Randomised trials and cohort studies of any type. Longitudinal studies. | USA: 8 Canada: 2 Australia: 3 UK: 2 France: 2 Finland: 1 Sweden: 1 Netherlands: 1 Germany: 1 Iran: 1 India: 1 More than one country: 1 | 6; medium |
| Bowness, 2023 (28) * | “To provide a comprehensive overview of participatory research conducted with carers.”  Barriers and facilitators. | 1998 – 2023. | (Unpaid) carers | 55  N = Not reported.  Unique studies 52 of 55. | 15 qualitative; 28 quantitative; 12 mixed methods. | Family/informal carers | Finland - 1 Ireland - 2 Australia - 7  Malawi - 1  USA - 13 Kenya - 1 UK - 18  Peru - 1 USA and Canada - 1 Korea - 1 Germany - 1 Canada - 4 New Zealand - 1 Sweden - 2 UK and Australia - 1 | 8; high |
| Bricca, 2022 (29) * | "To investigate predictors of participant eligibility, recruitment and retention in behavioural randomized controlled  trials for smoking cessation."  Barriers and facilitators. | 1996-2018. | Adults wanting to stop smoking. | 172  N = 89,639.  Unique studies 172 of 172. | 172 quantitative. | Smoking cessation | Denmark - 1  France - 2  Germany - 1  Netherlands - 4  Norway - 3  Spain - 4  Sweden - 1  Switzerland - 5  Turkey - 1  UK - 13  Australia - 7  New Zealand - 1  USA - 110  Canada - 7  China - 6  Hong Kong - 2  Japan - 1  South Korea - 1  Malaysia - 1  Pakistan - 1 | 7; medium |
| Bugeja, 2018 (30) * | “To identify and examine the barriers to and enablers of patient recruitment in chronic wound RCTs.”  Barriers and facilitators. | 2003 – 2017. | Adult patients (as defined by study authors) undergoing treatment for a skin ulcer, leg ulcer, varicose ulcer, and diabetic foot ulcer | 27  *N = 1,967.*  Unique studies 25 of 27. | 1 qualitative; 26 quantitative. | Adults undergoing treatment for skin ulcer, leg ulcer, varicose ulcer and diabetic foot ulcer | UK: 12 USA: 2 Canada: 1 Australia: 4 Germany: 1 India: 1 New Zealand: 1 Mexico: 1 Italy: 1 More than one country: 3. | 7; medium |
| Chambers, 2019 (31) * | “To examine the evidence regarding patient/carer involvement in palliative care research and identify the facilitators, barriers.”  Barriers and facilitators. | 2000 -2019. | Patient/carer involvement in palliative care | 60.    *N = 3,852.*  Unique studies 59 of 60. | Not reported. | Palliative care (range of research types) | UK 48; USA 6; Canada 1; Denmark 1; Australia 1; Ireland 1; Netherlands 1;  More than one country 1. | 7; medium |
| Chatland, 2023 (32) * | “To gain a deeper understanding of the experiences and perspectives of adult patients, carers and family members who have participated in a palliative and end-of-life care research study.”  Barriers and facilitators. | 2013 – 2018. | Adult patients, carers or family members with experience of participating in 'palliative and end-of-life care' research | 4  *N = 417.*  Unique studies 3 of 4. | 4 qualitative. | Palliative and end-of-life care | USA: 2  Switzerland: 1  Australia: 1 | 6; medium |
| Crane, 2017 (33) | “To examine ethical issues surrounding research with children and adolescents from their perspective as participants.”  Barriers and facilitators. | 2003–2014. | Children and adolescents, majority with physical or mental illness | 9 studies  (23 in full review)  *N* = 6326  Unique studies: 8 of 9 | 4 qualitative; 3 quantitative; 2  mixed methods. | Vaccine trials (any Phase). | Sweden: 2  USA: 7 | 5, medium |
| Dean, 2023 (34) * | "To understand why individuals do or do not take part in vaccine trials.”  Barriers and facilitators. | 1999 – 2021. | Participants invited to take part in vaccine trials | 34  N = 11,650 (excluding one study that did not report participant numbers).  Unique studies 15 of 34. | 19 qualitative; 12 quantitative; 3 mixed methods. | Vaccine trials (Phases 1-4). | USA: 9  South Africa: 1  Peru: 1  Canada: 4  UK: 2  Spain: 1  France: 1  Kenya: 3  Guinea: 1  Sierra Leone: 2  Finland: 1  Papua New Guinea: 1  Tanzania: 2  India: 1  Netherlands: 1  More than one country: 3. | 8; high |
| Dhalla, 2013 (35) | “To review barriers to participation in actual preventative HIV vaccine trials.”  Barriers only. | 1994 –2012. | Adults  8 studies involving low-risk adults  12 studies involving ‘higher risk’ adults (e.g. intravenous drug users, gay men, sex workers) | 20 studies  *N* = 18,033  Unique studies: 8 of 20 | Not reported. | HIV vaccine trials (any Phase). | Canada: 1  Kenya: 1  Spain: 1  Tanzania: 2  Thailand: 4  UK: 1  USA: 8  Multiple countries: 1  Not reported: 1 | 7, medium |
| Dhalla, 2014 (36) | “To better understand motivators to participation in actual preventive HIV vaccine trials.”  Facilitators only. | 1997–2011 | Adults.  9 studies involving low-risk adults  5 studies involving ‘higher risk’ adults (e.g. intravenous drug users, gay men, sex workers)  6 studies involving a mix of high- and low-risk adults  1 study unknown risk | 21 studies  *N* = 32,825  Unique studies: 11 of 21 | Not reported. | HIV vaccine trials (any Phase). | Canada: 1  Italy: 1  Spain: 1  Tanzania: 2  Thailand: 6  UK: 1  USA: 6  Multiple countries: 3 | 6, medium |
| Escritt, 2022 (37) * | “To synthesise the qualitative evidence regarding cancer patient experiences during Phase 1 trials from recruitment to post-trial follow-up.”  Barriers and facilitators. | 1984 – 2020. | Adults with cancer enrolled in phase 1 trials. | 13 studies    N = 328.  Unique studies 5 of 13. | 9 qualitative; 4 mixed methods. | Cancer drug trials (Phase 1). | France: 1  USA: 8  Sweden: 1  Japan: 1  UK: 1  Netherlands: 1 | 8; high |
| Fayter, 2007 (38) | “To undertake a systematic review of the relevant literature relating to the barriers, modifiers, and benefits involved in participating in RCTs of cancer therapies.”  Barriers and facilitators. | 1996–2004. | Patients (adults and children) diagnosed with various cancers | 37 studies (56 in full review)  *N* = 25,788 (plus an unreported number from 4 studies)  Unique studies: 16 of 37 | 5 qualitative; 31 quantitative; 1 mixed methods. | Randomised controlled trials (excluding solely phases I or II) | Australia: 3  Canada: 1  Denmark: 1  Finland: 1  Italy: 1  Netherlands: 1  Sweden: 1  UK: 12  USA: 15  Multiple countries: 1 | 8, high |
| Ferguson, 2023 (39) * | "To describe perspectives about clinical trial participation, including barriers and  facilitators for trial recruitment and retention among individuals with pain-related conditions, depression, and/or anxiety. "  Barriers and facilitators. | 2007-2020. | Individuals with pain and pain comorbidities, including depression and/or anxiety. | 34 studies.  N = 6,990 (one study sample size not reported).  Unique studies 28 of 34. | 14 qualitative; 15 quantitative; 5 mixed methods. | Treatment trials in pain, depression and/or anxiety. | UK 14; USA 10; Australia 2; Italy, Germany, Netherlands, New Zealand, China 1 each; More than one country 3. | 5; medium |
| Fisher, 2011 (41) | “This review considers the reasons that parents accept or decline an invitation to enrol children of any age in clinical research.”  Barriers and facilitators. | 2001–2011 | Parents/caregivers of children invited to take part in research  Two thirds of studies involved children with life-limiting or life-threatening conditions including cancer and diabetes | 16 studies  *N* = 365 (plus an unreported number from one study).  Unique studies: 7 of 16 | 16 qualitative. | Children in clinical research. | Canada: 1  Gambia: 1  Malawi: 1  UK: 4  USA: 9 | 7, medium |
| Fisher, 2020 (40) * | "To investigate the role of race and ethnicity in views toward and participation in genetic studies and precision medicine research in the United  States."  Barriers and facilitators. | 2006-2018. | Ethnic minority populations in the United States. | 27 studies.  N = 145,379 (Including white people). Ethnic minority participants N = 42,958.  Unique studies: 26 of 27. | 8 qualitative; 19 quantitative. | Views of ethnic minorities towards genetic studies and precision medicine. | USA: 27 | 6; medium |
| Forcina, 2018 (42) | “To conduct a systematic review of studies limited to adolescent and young adult patients which assessed attitudes and beliefs that influence cancer clinical trial enrolment.”  Barriers and facilitators. | 2009–2016 | Adolescent and young adult cancer patients aged 15–39 years | 6 studies  *N* = 754  Unique studies: 3 of 6 | 3 qualitative; 3  quantitative. | Cancer clinical trials. | USA: 1  Not reported: 5 | 6, medium |
| Ford, 2008 (43) * | “We performed a systematic review of barriers to participation of underrepresented populations in controlled trials of cancer prevention or treatment.”  Barriers and facilitators. | 1984 – 2006. | Under-represented populations involved in cancer related trials | 57 (out of 65)  N = 608,441. (2 studies not reported).  Unique studies 38 of 57. | 24 qualitative; 33 quantitative. | Prevention, treatment and survivorship cancer clinical trials. | Not reported | 7; medium |
| Forsat, 2020 (44) * | " To identify barriers and solutions for the  recruitment and retention of older (aged >65 years) people in clinical trials."  Barriers and facilitators. | 1991-2019. | Older adults (>65 years old) | 50.  *N = 407,954*  Unique studies 42 of 50. | Not reported. | Older adults (>65 years) in clinical research. | Australia: 6  USA: 26  UK: 7  Netherlands: 2  Canada: 1  New Zealand: 2  Denmark: 2  Norway: 2  France: 1  More than one country: 1 | 6; medium |
| Furyk, 2018 (45) * | “To review the available empiric evidence about alternatives to prospective informed consent in the context of paediatric acute care research.”  Barriers and facilitators. | 2004 - 2017. | Children, families, health care staff, institutions, and the community involved in paediatric acute care research | 13 (out of 15)  *N = At least 5,199. (In Molyneux, 2013 unclear how many focus group participants).*  Unique studies 13 of 13. | 6 qualitative; 5 quantitative; 2 mixed methods. | Paediatric acute care research. | USA: 4 UK: 7 More than one country: 2 | 8; high |
| Gad, 2018 (46) | “To determine the factors that influence *[cancer]* patients’ decisions to enter a phase I trial.”  Barriers and facilitators. | 1995–2014 | Adult patients diagnosed with various cancers | 15 studies (37 in full review).  *N* = 1,313.  Unique studies: 7 of 15 | 4 qualitative; 11  quantitative. | Trials (Phase I). | Canada: 1  Italy: 1  Japan: 2  UK: 3  USA: 8 | 10, high |
| Gilmore-Bykovskyi, 2019 (47) * | "To systematically review the literature for published reports describing recruitment and  retention of individuals from underrepresented backgrounds in Alzheimer's disease and related dementias research or underrepresented  participants’ perspectives regarding ADRD research participation. "  Barriers and facilitators. | 2010-2017. | Ethnic minority groups involved in Alzheimer's disease and related dementias research | 22    *N = 5,794*  Unique studies 16 of 22. | 6 qualitative; 16 quantitative. | Alzheimer's disease and related dementias research. | USA 20 UK 1. 1 not reported. | 8; high |
| Glover, 2015 (48) | “A systematic review of the literature on challenges and facilitators of participation in health RCTs amongst Indigenous people from New Zealand, Australia, Canada and the USA.”  Barriers and facilitators. | 1994–2011 | 5 studies with Indigenous or Aboriginal Australians, 4 studies with First Nation participants, 6 studies with Maori participants, 31 studies with Native Americans or Alaskan Natives  The majority (*N* = 11) focused on cancer, 7 on diabetes, 6 on substance abuse and 22 on other conditions/factors | 46 studies  *N* = not reported  Unique studies: 43 of 46 | Not reported. | Randomised controlled trials. | Australia: 5  Canada: 4  New Zealand: 6  USA: 31 | 5, medium |
| Goldstein, 2021 (49) * | “To synthesize the existing knowledge on recruitment and retention of pregnant women in birth cohort studies.”  Barriers and facilitators. | 1992-2019. | Pregnant women involved in prospective birth cohort studies | 38  *N = 63,014.*  Unique studies 36 of 38. | Not reported. | Birth cohort studies. | USA: 16  Canada: 6  Australia: 4  UK: 4  Brazil: 1  Netherlands: 1  Lebanon: 1  Oman: 1  Italy: 2  China: 1  New Zealand: 1 | 5; medium |
| Gorzynska, 2022 (50) * | “To systematically review qualitative studies exploring the experiences of participating in clinical trials from the perspectives of patients with Parkinson's disease and their caregivers.”  Barriers and facilitators. | 2005-2020. | Patients with an established Parkinson's Disease diagnosis | 11.    N = 378.  Unique studies 10 of 11. | 11 qualitative. | Trials in Parkinson's disease. | UK: 4  Jordan: 1  USA: 4  Australia: 1  Netherlands: 1 | 6; medium |
| Grand, 2012 (51) | “This review examines the relationship between the obstacles to participation in cancer clinical trials and accrual.”  Barriers only. | 1983–2007 | Patients diagnosed with various cancers | 20 studies (31 in full review)  *N* = 13,681  Unique studies: 10 of 20 | 6 qualitative; 14 quantitative. | Oncology clinical trials. | Not reported | 5, medium |
| Gregersen, 2019 (52) | “To systematically review and thematically synthesise the experiences of patients and relatives when they have to decide whether or not to participate in a clinical oncology trial and to provide knowledge about the decision-making process.”  Barriers and facilitators. | 2000–2016 | Adult patients with advanced cancer | 11 studies  *N* = 203  Unique studies: 4 of 11 | 11 qualitative. | Clinical trials. | USA: 6  UK: 3  Japan: 1  Sweden: 1 | 5, medium |
| Hasan, 2021 (53) * | "To describe stakeholder perspectives regarding research studies involving children with cancer at the EOL *(end of life).*“  Barriers and facilitators. | 1977-2020" | Children with incurable cancer. | 15 (out of 24)  *N = 941.*  Unique studies 5 of 15. | 10 qualitative; 5 quantitative. | End of life paediatric cancer trials. | USA 10; Netherlands 2; Canada 1; More than one country 1; 1 not stated. | 7; medium |
| Hosie, 2022 (54) * | “To identify the perspectives and experiences of older persons and their caregivers of research participation with impaired decision-making capacity.”  Facilitators only. | 2000 - 2020 | Older persons with impaired decision-making capacity and their caregivers. | 23  N = 7,331.  Unique studies 21 of 23. | 4 qualitative; 19 quantitative. | Any research topic. | US: 19  Canada: 2  Australia: 1  Israel: 1 | 4; medium |
| Houghton, 2020 (55) * | “To explore potential trial participants' views and experiences of the recruitment process for participation.”  Barriers and facilitators. | 2004-2018 | Adult participants invited to participate in randomised controlled trials. | 29.  *N = 847.*  Unique studies 14 of 29. | Not reported. | Randomised trials (medicine, mental health, oncology, surgery, pregnancy and childbirth, and health promotion). | UK: 16  USA: 3  Australia: 1  Canada: 1  New Zealand: 1  Tanzania: 1  Austria: 1  Denmark: 1  Germany: 2  Sweden: 1  Netherlands: 1 | 7; medium |
| Hughes-Morley, 2015 (56) | “To systematically identify relevant qualitative studies describing factors affecting recruitment of participants into depression trials.”  Barriers and facilitators. | 2007–2012 | Patients with depression | 4 studies (15 in full review)  *N* = 1,034  Unique studies: 1 of 4 | 4 qualitative. | Randomised controlled trials. | UK: 3  Multiple countries: 1 | 9, high |
| Le, 2023 (57) * | "To identify barriers and strategies  associated with increasing recruitment of visible minorities in MSD research."  Barriers and facilitators. | )  1997-2022. | Patients with musculoskeletal disorders of 'visible minorities'. | 28  *N = 13,226.*  Unique studies 23 of 28. | 9 qualitative; 19 quantitative. | Musculoskeletal health research. | USA - 27  Australia - 1 | 5; medium |
| Le Rouzic, 2020 (58) * | “To thoroughly describe the process of parental decision-making regarding their children's participation in Phase I oncology trials.”  Barriers and facilitators. |  | Parents of children with cancer | 18 (out of 30)  *N = 363.*  Unique studies 10 of 18. | 12 qualitative; 4 quantitative; 2 mixed methods. | Children’s oncology trials (Phase 1). | USA 12; Mexico 1; Switzerland 1; Canada 1; France 2; More than one country 1; | 4; medium |
| Liljas, 2017 (59) | To identify facilitators, barriers and strategies for engaging ‘hard to reach’ older people in research on health promotion; the oldest old (≥ 80 years), older people from black and minority ethnic groups (BME) and older people living in deprived areas.”  Barriers and facilitators. | 1996–2014. | 18 studies of BME older people (aged over 50 years), 3 studies with oldest old patients (80 years and over) and 2 studies of older people in deprived areas | 23 studies  *N* = not reported  Unique studies: 23 of 23 | 12 qualitative; 10  quantitative; 1  mixed methods. | Not reported. Studies focused on health promotion | Canada: 1  New Zealand: 1  UK: 4  USA: 17 | 7, medium |
| Lima, 2020 (60) * | “To identify perceptions of patients from historically underrepresented groups on participation in rheumatology-related research studies.”  Barriers and facilitators. | 2004 - 2018 | Adult patients with rheumatic disease | 7  *N = 2,072.*  Unique studies 2 of 7. | 2 qualitative; 5 quantitative. | Under-represented groups in rheumatology-related research studies. | US 7 | 7; medium |
| Limkakeng, 2013a (61) | “To understand Chinese patients’ motivations and concerns to participate in clinical trials.”  Barriers and facilitators. | 2004–2008 | Chinese adults between 18 years and 85 years  1 study relating to HIV vaccine trials and 1 relating to cancer, 3 studies non-specific | 5 studies  *N* = 645.  Unique studies: 4 of 5 | 3 qualitative; 2 quantitative. | Clinical trials. | USA: 3  China: 1  Singapore: 1 | 8, high |
| Limkakeng, 2013b (62) | “To conduct a systematic review and meta-summary to evaluate what values, attitudes or beliefs on the part of potential or actual research participants with emergent medical conditions influence participation in research.”  Barriers and facilitators. | 2000–2009 | Adult patients aged over 18 years  5 studies focused on suspected myocardial infarction patients, 3 on stroke patients, 1 on sudden cardiac near-death survivors and 5 on other emergency patients | 14 studies  *N* = 4,003 (plus 1 study unclear)  Unique studies: 12 of 14 | 6 qualitative; 8 quantitative; 3  mixed methods. | Not reported. | Primarily conducted in American and Western European contexts. Number not reported | 7, medium |
| Lond, 2024 (63) * | “To summarize the factors and contexts that influence cancer patient decision making related to clinical trial participation.”  Barriers and facilitators. | Studies 2006-2023. | Patients with lung cancer | 18 studies  *N = 14,153.*  Unique studies 15 of 18. | 8 qualitative; 10 quantitative. | Lung cancer treatment trials. | USA 14; Canada 1; UK 1; Denmark 1. | 8; high |
| Lovell, 2023 (64) * | “To investigate factors which influence participation in maternity research for women from an ethnic minority background.”  Barriers and facilitators. | 2005-2022. | Pregnant women | 14 studies.  *N = 12,073, but only data regarding minority ethnic women were extracted, of which N = 6,339.*  Unique studies 10 of 14. | 1 qualitative; 9 quantitative; 4 mixed methods. | Pregnant women in ethnic minority groups. | USA - 11  UK - 3 | 8; high |
| Martinsen, 2016 (65) | “To perform a systematic review of the current literature on participation motives, response rates and recruitment  strategies in research bronchoscopy studies with an emphasis on studies including COPD patients.”  Barriers and facilitators. | 1998–2013 | Patients with HIV, bronchoscopy patients, smokers, children with or without cystic fibrosis and parents | 6 studies (7 in full review)  *N* = 455  Unique studies: 6 of 6 | Not reported. | Not reported. | The Netherlands: 1  UK: 3  USA: 1  Multiple countries: 1 | 5, medium |
| Matthews, 2023 (66) * | “To explore the proportional representation of women in contemporary cardiovascular research and the factors (barriers and enablers) that affect their participation in cardiovascular studies.”  Barriers and facilitators. | 2013-2020. | Female adults involved with medical or interventional cardiovascular disease treatment trials. | 10  *N = 1,211,366.*  Unique studies 6 of 10. | 1 qualitative; 9 quantitative. | Cardiovascular disease treatment trials. | UK - 1  USA - 8  Canada - 1 | 6; medium |
| McCann, 2007 (67) | “To draw together qualitative and quantitative studies reporting patients’ experiences of trial recruitment and participation to provide a broad-based overview of the literature.”  Barriers and facilitators. | 1982–2005 | Demographic data largely not reported. Range of trials including HIV, cancer, neonatal and myocardial infarction | 32 studies  *N* = 6,068  Unique studies: 20 of 32 | 12 qualitative; 12 quantitative; 3  mixed methods. | Trials (Phase III). | Denmark: 2  Europe: 1  Israel: 1  UK: 13  USA: 9  The Netherlands: 1  Multiple countries: 5 | 4, medium |
| McCann, 2013 (68) | Update of McCann (2007) review—no new objective stated.  Barriers and facilitators. | 2006–2010 | Patients with a variety of conditions including cancer, epilepsy, stroke and pre-term labour. One paper discussed interviews with parents of children with leukaemia and 2 studies involved pregnant women or parents | 11 studies (12 in full review)  *N* = 290.  Unique studies: 3 of 11 | Not reported. | Trials. | Australia: 1  Denmark: 1  UK: 7  USA: 1  Multiple countries: 1 | 7, medium |
| Meskell, 2023 (69) * | “To explore the factors that influence a person's decision to participate in a vaccine trial in the context of a pandemic or epidemic.”  Barriers and facilitators. | 2001-2021 | Adults invited to take part in a vaccine trial in the context of an epidemic or pandemic. | 34  *N = 5,449.*  Unique studies 22 of 34. | 34 qualitative. | Vaccine trials in the context of a pandemic or epidemic | USA 16; India 1; Canada 4; Guinea 1; Sierra Leone 2; South Africa 1; Tanzania 4; Kenya 1; Democratic Republic of Congo 1; More than one country 3. | 9; high |
| Mills, 2006 (70) * | “To assess studies of barriers to participation in experimental trials and randomised trials for validity and content.”  Barriers only. | 1982-2005 | Adults involved in clinical trial participation | 33    *N = 6,174.*  Unique studies 12 of 33. | 12 qualitative; 21 quantitative. | Oncology trials. | Not reported | 8; high |
| Moffat, 2023 (71) * | “To identify the factors associated with the recruitment of individual patients, practices or practitioners to RCTs in general practice.”  Barriers and facilitators. | 1999-2018 | Patients and practitioners in primary care invited to participate in RCTs. | 37 studies.  *N = 21,153.*  Unique studies 32 of 37. | 10 qualitative; 17 quantitative; 10 mixed methods. | Randomized trials in general practice. | UK: 18  USA: 5  Australia: 5  New Zealand: 3  Netherlands: 3  Germany: 1  Norway: 1  Switzerland: 1 | 8; high |
| Naidoo, 2020 (72) * | "To explore research burdens and benefits  of adult patients’ participation in RCTs. This should allow the subsequent improvement of research planning and conduct."  Barriers and facilitators. | 2001-2017 | Patient participants of RCTs. | 45 studies.  N = Not reported.  Unique studies: 27 of 45. | 45 qualitative. | Randomised control trials in cancer and chronic diseases. | Not reported. | 4; medium |
| Nalubega, 2015 (73) | “To synthesize and present the best available evidence in relation to HIV research participation in sub-Saharan Africa, based on the views and experiences of research participants.”  Barriers and facilitators. | 2004–2014. | All current or former adult HIV research participants from sub-Saharan African countries. 16 studies only involved women | 21 studies.  *N* = not reported.  Unique studies: 16 of 21 | 21 qualitative: | Not reported. | Kenya: 1  Malawi: 1  South Africa: 12  Tanzania: 4  Zimbabwe: 1  Multiple countries: 2 | 9, high |
| Natale, 2021 (74) * | “To describe patient perspectives on recruitment and retention in clinical trials.”  Barriers and facilitators. | 1998-2019 | Patients ages 18 or over with any health condition that had been invited to participate in clinical trials. | 63 studies.    *N = 1,681*  Unique studies 38 of 63. | 63 qualitative. | Clinical trials. | Australia: 5; Brazil: 1; Canada: 5; China: 1; Denmark: 1; Germany: 1; Japan: 2; Russia: 1; Singapore: 1; Sweden: 1; UK: 12; US: 32. | 5; medium |
| Nathe, 2023 (75) * | To assess barriers and facilitators to paediatric research participation and evaluate differences by enrolment status.  Barriers and facilitators. | 1954-2020 | Parents of children that were asked to participate in paediatric research | 70 studies.    *N = Not reported.*  Unique studies 61 of 70. | 22 qualitative; 46 quantitative; 2 mixed methods. | Paediatric research. | US: 26  Canada: 14  UK: 10  Australia: 5  Italy: 2  New Zealand: 1  Sweden: 1  Denmark: 1  Netherlands: 1  Germany: 1  Ireland: 1  India: 1  Egypt: 1  Tanzania: 1  Finland: 1  France: 1  More than one country: 2 | 7; medium |
| Neelakantan, 2023 (76) * | "To locate and summarise  the limited research on adolescents’ experiences of participating in research on sensitive topics."  Barriers and facilitators. | 2008-2020. | Adolescents asked to take part in research into sensitive subjects. | 17 studies.  *N = 3,414.*  Unique studies 17 of 17. | 9 qualitative; 8 mixed methods. | "'Sensitive research, namely HIV/AIDS, violence against children, drug use and substance  abuse, sexuality and sex-related topics, including LGBTQ issues." | UK 6;  Australia 3;  South Africa 3;  USA 2;  Uganda 1;  Belgium 1;  More than one country 1. | 5; medium |
| Nielsen, 2019 (77) | “To examine cancer patients’ perceptions of factors that may influence their decisions on participation in phase I–III clinical drug trials.”  Barriers and facilitators. | 2010–2013 | Adult cancer patients | 9 studies  *N* = 236  Unique studies: 1 of 9 | 9 qualitative. | Cancer clinical drug trials. | USA: 7  Japan: 1  Sweden: 1 | 5, medium |
| Nievaard, 2004 (78) | “To assess the factors that may influence a patient’s consent to participate in a clinical trial.”  Barriers and facilitators. | 1984–2002 | Adult patients. 14 with cancer patients, 5 with HIV patients, 6 from other patient groups and 5 did not report the patient group | 30 studies  *N* = not reported  Unique studies: 18 of 30 | Not reported. | 6 randomised controlled trials, others not reported. | Australia: 3  USA: 14  Western Europe: 13 | 5, medium |
| Nobile, 2013 (79) | “To review the literature addressing actual and apparently healthy participants’ reasons to enrol in biobank studies in order to see if some motives are unduly influencing the decision to participate.”  Facilitators only. | 2006–2012 | Healthy adult participants.  4 studies involved just women | 13 studies  *N* = 1,762  Unique studies: 11 of 13 | 9 qualitative; 4 quantitative. | Not reported. | Australia: 2  Europe: 1  UK: 3  USA: 7 | 5, medium |
| Phelps, 2020 (80) * | “To understand patients' experiences of recruitment and participation in surgical trials and identify barriers and facilitators to participation in surgical trials.”  Barriers and facilitators. | 1998-2018. | Adults involved in surgical RCTs. | 34 studies.  *N = 3,294.*  Unique studies 24 of 34. | 21 qualitative; 11 quantitative; 2 mixed methods. | Experience of adult patients in surgical RCTs | UK 20  USA 5  Sweden 2  Canada 1  Denmark 2  Australia 1  Japan 1.  More than one country: 2 | 7; medium |
| Prescott, 1999 (81) | “To assemble and classify a comprehensive bibliography of factors limiting the quality, number and progress of RCTs.”  Barriers only. | 1986–1996 | Majority of studies involved cancer patients (*N* = 9), 2 studies concerning child health involved parents/caregivers | 22 studies (27 studies in chapter)  *N* = 15,295  Unique studies: 18 of 22 | Not reported. | Clinical trials (not Phases I or II). | Australia: 1  Canada: 1  France: 3  The Netherlands: 1  UK: 5  USA: 11 | 8, high |
| Quay, 2017 (82) | “To identify barriers and facilitators to recruitment of South Asians to health research studies and associated strategies to improve participation.”  Barriers and facilitators. | 2004–2016 | South Asian patients. Majority of studies involved patients with a condition, e.g. asthma or diabetes | 10 studies (15 in full review)  *N* = 3,139  Unique studies: 8 of 10 | 6 qualitative; 3 quantitative; 1 mixed methods. | Randomised control trials. | Australia: 1  India: 1  UK: 7  USA: 1 | 8, high |
| Raimundo-Silva, 2024 (83) * | "To increase the understanding of the reasons for and against participating in biomedical research on neglected tropical diseases."  Barriers and facilitators. | 2009-2021. | People at risk of neglected tropical diseases (WHO list of 20 diseases). | 11 studies.    *N = Not reported.*  Unique studies 11 of 11. | Not reported. | Interventions research into neglected tropical diseases. | Ethiopia 5; Uganda 2; Puerto Rico 2; Indonesia 1; Sudan 1. | 7; medium |
| Sedrak, 2021 (84) * | “We conducted a systematic review focused on evaluating 2 questions: 1) What barriers hinder participation of older adults in cancer clinical trials? and 2) What interventions influence and improve their participation beyond trials designed specifically for their age group?”  Barriers and facilitators. | 2002-2018 | Adults with cancer | 13 studies.  *N = Not reported.*  Unique studies 8 of 13. | 13 quantitative. | Cancer treatment trials. | USA 8; Canada 2; Netherlands 1; Ireland 1; Germany 1. | 5; medium |
| Townsley, 2005 (85) * | "To identify the barriers that impede the accrual of this vulnerable population onto clinical trials.”  Barriers only. | 1994 - 2004 | Older patients (adults over 65 years old) | 9 studies.  *N = Not reported.*  Unique studies 4 of 9. | Not reported. | Older patients and clinical trials. | USA - 8 Canada - 1 | 5; medium |
| Tromp, 2016 (86) | “To answer the following research question: What are motivating and discouraging factors for children and their parents to decide to participate in clinical drug research?”  Barriers and facilitators. | 1997–2013 | 26 studies involved parents or caregivers/guardians, 5 involved children and 11 involved both. Included children aged between 6 and 21  Diverse research population but many involved oncology patients (11 studies)  39 studies involved people who had consented, 24 involved people who had dissented. 29 studies involved treatments with prospect of direct benefit | 42 studies  *N* = 5,500  Unique studies: 28 of 42 | 16 qualitative;  26 quantitative. | Not reported | Not reported | 7, medium |
| Van der Zande, 2018 (87) | “To systematically review all articles regarding pregnant women’s reasons to participate in clinical research.”  Barriers and facilitators. | 2013–2016. | Pregnant/previously pregnant women | 30 studies  *N* = 7,905, plus an unreported number from 1 study.  Unique studies: 26 of 30 | Not reported. | Observational studies and randomised controlled trials | UK: 10  USA: 7  Canada: 5  Australia: 2  China: 1  Ghana: 1  Ireland: 1  Italy: 1  Netherlands: 1  Pakistan: 1 | 5, medium |
| Van Lent, 2021 (88) * | "To synthesize and describe which patient values play a role in the decision-making process for early phase clinical trials. We also aim to indicate how these factors relate to the decision to participate or not in early phase clinical trials."  Barriers and facilitators. | 2002 - 2020 | Adult smokers | 16 studies.  N = 2,046.  Unique studies 7 of 16. | 5 qualitative; 11 quantitative. | Cancer trials (Phases I or II). | USA: 12  UK: 2  Netherlands: 1  Japan: 1. | 7; medium |
| Viljoen, 2020 (89) * | "To synthesise literature reporting  experiences of participants when deciding to enrol in a cancer clinical trial in order to inform practice."  Barriers and facilitators. | 1999-2018. | People with cancer | 40 studies.  *N = 1,531.*  Unique studies 15 of 40. | 40 qualitative. | Cancer trials | USA 21; UK 11; Netherlands 2; Sweden 2; Denmark 2; Singapore, Canada, 1 each. | 8; high |
| Weaver, 2019 (90) * | “To report the benefits and burdens of palliative research participation on children, siblings, parents, clinicians, and researchers.”  Barriers and facilitators. | 2002-2017. | Child involved in paediatric palliative care research and their siblings, parents, clinicians, or researchers. | 22 studies (out of 24).  *N = 2,070.*  Unique studies 22 of 22. | 10 qualitative; 6 quantitative; 3 mixed methods.  1 literature review. | Paediatric palliative care research. | USA 12 Australia 4 Sweden 2 Canada 1 Norway 1 More than one country 2 | 7; medium |
| Woodall, 2010 (91) | “To review the current literature on the nature of barriers to participation across different mental health studies with a focus on whether there are specific gender-, age- and ethnicity-related barriers.”  Barriers only. | 1992–2008. | Adult participants.  5 schizophrenia studies, 5 depression studies, 6 dementia studies and 5 where the illness was not specified | 16 studies (49 in full review)  *N* = 2,033, plus an unreported number from 9 studies  Unique studies: 14 of 16 | Not reported. | Not reported. | Australia: 1  Canada: 1  Germany: 1  Mexico: 1  Switzerland: 1  UK: 1  USA: 10 | 6, medium |
| Xiao, 2022 (92) * | “To explore the factors influencing patient participation in cancer clinical trials.”  Barriers and facilitators. | 2011-2019. | Cancer patients | 40  *N = Not reported.*  Unique studies 27 of 40. | 15 qualitative; 20 quantitative.  Other studies not reported. | Cancer clinical trials. | USA 20; UK 5; Australia 4; Not reported 11. | 5; medium |

(The 44 systematic reviews newly included in the review update have been marked with an asterisk * in the first column)
