# Supplementary material for: Why do patients take part in research? An updated overview of systematic reviews of psychosocial barriers and facilitators
Source: Trials. 2025 May 27;26:174. doi: 10.1186/s13063-025-08850-6 (PMC12107899; doi:10.1186/s13063-025-08850-6)
Supplement: Supplementary file 2 — Supplementary Materials Table 2. Barriers and facilitators identified in systematic reviews, mapped to the Theoretical Domains Framework and recruitment interventions. [file 13063_2025_8850_MOESM2_ESM.docx]

**Supplementary Materials Table 2. Barriers and facilitators identified in systematic reviews, mapped to the Theoretical Domains Framework and recruitment interventions.**

| **Identified theme** | **Systematic reviews reporting the theme** | **Domain (components) of the TDF (Cane et al., 2012)** | **From Parker, Treweek et al., (in preparation): Interventions that probably affect research recruitment.** | **From Parker, Treweek et al., (in preparation): Interventions with uncertain effects on research recruitment.** | **From Parker, Treweek et al., (in preparation): Interventions shown not to affect research recruitment.** |
| --- | --- | --- | --- | --- | --- |
| **FACILITATORS** |  |  |  |  |  |
| **Altruism (including benefits to science; helping others)** | Reported in 52 SRs: (24, 26, 27, 31, 32, 34, 36-43, 45, 47, 49, 52, 54-56, 58, 60-69, 71-76, 78, 80, 82, 83, 85-88, 90, 92) | Beliefs about consequences (Reflective Motivation) |  | Letter stressing ethnicity-specific disease. |  |
| **Personal or family benefit (including therapeutic benefits; closer monitoring; access to new treatments; gaining knowledge of own health or new skills)** | Reported in 48 SRs: (24, 26, 27, 32, 34, 35, 37-43, 47, 49-56, 58-63, 65-69, 73-80, 82, 83, 85, 86, 88, 92) | Optimism (Reflective Motivation)  or  Environmental context and resources (Physical Opportunity) | Mentioning scarcity of trial places. | Positive framing of potential treatment benefits.  Letter stressing ethnicity-specific disease. | Patient preference trial design. |
| **Trust or confidence in the physician or the research** | Reported in 34 SRs: (31, 33, 34, 39, 40, 43, 45, 47, 51, 52, 56, 58-65, 67-69, 72, 74-80, 83, 88, 90, 92) | Professional Role / Identity (Reflective Motivation)  or  Social Influences (Social Opportunity) |  | Endorsements of previous participants.  SMS messages with quotes from existing participants.  Total information disclosure. |  |
| **Convenient or low burden research** | Reported in 13 SRs: (25, 39, 47, 49, 54, 61, 64, 69, 75, 79, 83, 86, 87) | Belief about consequences (Reflective Motivation)  or  Environmental context and resources (Physical Opportunity) |  | Opt-out consent method.  Paper data capture. | Pen provision. |
| **Financial or practical incentives** | Reported in 11 SRs: (34, 43, 47, 49, 61, 66, 73, 75, 76, 83, 86) | Goals (Reflective Motivation)  or  Environmental context and resources (Physical Opportunity) |  | Financial incentives. |  |
| *** Feeling valued** | Reported in 7 SRs: (25, 26, 28, 31, 54, 83, 89) | Reinforcement (Automatic Motivation) or  Social Influences (Social Opportunity) |  | Endorsements of previous participants.  PPI-designed participant information.  SMS messages with quotes from existing participants.  Handwritten personalised letter. | Leaflet stressing PPI involvement.  Educational programme on minority recruitment. |
| *** Opportunity for interaction with peers or healthcare professionals (including learning from others)** | Reported in 6 SRs: (25, 31, 50, 76, 83, 90) | Social Influences (Social Opportunity) |  | Endorsements of previous participants. |  |
| *** Similarity of staff to patients, or community representativeness** | Reported in 5 SRs: (39, 47, 63, 83, 89) | Social Influences (Social Opportunity) |  | SMS messages with quotes from existing participants. |  |
| *** Sense of duty or obligation** | Reported in 3 SRs: (55, 69, 76) | Beliefs about consequences (Reflective Motivation) |  | Letter stressing ethnicity-specific disease. |  |
| *** Personal relevance of the research** | Reported in 3 SRs: (28, 49, 90) | Reinforcement (Automatic Motivation) |  | Decision aid about trials.  Letter stressing ethnicity-specific disease.  Gender-targeted recruitment postcard.  Handwritten, personalised letter. | Educational programme on minority recruitment. |
| *** Dissemination of results** | Reported in 3 SRs: (25, 47, 83) | Beliefs about consequences (Reflective Motivation) or  Reinforcement (Automatic Motivation) |  |  |  |
| *** Raising public or practitioner awareness of condition** | Reported in 2 SRs: (27, 32) | Beliefs about consequences (Reflective Motivation) or  Professional Role / Identity (Reflective Motivation) |  | Letter stressing ethnicity-specific disease.  Pre-engagement with potential trial sites. | Teaser campaign (via postcards). |
| *** Spontaniety** | Reported in 1 SR: (71) | Memory, attention and decision making (Psychological Capability) |  |  |  |
| *** Inclusion of preference arm in trial** | Reported in 1 SR: (80) | Reinforcement (Automatic Motivation) |  |  | Patient preference trial design. |
| *** Feeling of nothing to lose** | Reported in 1 SR: (55) | Reinforcement (Automatic Motivation) |  |  |  |
| **BARRIERS** |  |  |  |  |  |
| **Fear or perceived risk (to health, of experimental treatment or adverse effects; to personal consequences)** | Reported in 35 SRs: (34, 35, 37, 39, 41, 42, 45, 47, 51, 53, 56, 57, 60, 63-65, 68-70, 72-74, 77, 78, 80, 82-84, 86-89, 91, 92) | Belief about consequences (Reflective Motivation) | Emphasising pain in information (−) | Emphasising risk in information.  Positive framing of treatment effect. |  |
| **Practical difficulties (including additional procedures or appointments; transport; costs; work or caring responsibilities; other commitments)** | Reported in 32 SRs: (27, 30, 31, 38, 39, 42, 44, 47, 48, 51, 53, 56, 59, 60, 63, 65-67, 69, 71, 72, 74-76, 81, 82, 84, 86, 87, 90-92) | Belief about consequences (Reflective Motivation) |  | Financial incentives.  Digital data capture  (−). | Email (not postal) invitations. |
| **Distrust of research or researchers** **(particularly amongst ethnic minorities), including perceived threat to privacy, confidentiality or safety** | Reported in 26 SRs: (23, 27, 34, 40, 47, 48, 56, 57, 61-64, 67, 69, 71, 73-76, 80, 82-84, 86, 87, 91) | Reinforcement (Automatic Motivation) or  Social Influences (Social Opportunity)  or  Belief about Consequences (Reflective Motivation) |  | Endorsements of previous participants.  SMS messages with quotes from existing participants.    Total information disclosure.  Letter stressing ethnicity-specific disease.  Providing information in-person. | Leaflet stressing PPI involvement.  Educational programme on minority recruitment. |
| **Aversion to randomisation** | Reported in 17 SRs: (24, 39, 42, 56, 63, 66-68, 71, 74, 75, 78, 80, 85-87, 92) | Environmental context and resources (Physical Opportunity) | Open trial design | Total information disclosure.    Opt-out consent.    Multimedia education about trials (DVD + booklet). |  |
| **Treatment preference (for specific therapy; for standard care; against placebo)** | Reported in 16 SRs: (23, 29, 30, 38, 39, 51, 60, 64, 67, 80, 81, 83-86, 92) | Reinforcement (Automatic Motivation) | Open trial design | Opt-out consent.    Decision aid about trials.  Multimedia education about trials (DVD + booklet). | Patient preference trial design. |
| **Personal health (or health of child or other relative)** | Reported in 11 SRs: (25, 44, 53, 56, 59, 60, 62, 70, 83, 91, 92) | Emotion (Automatic Motivation) |  | Letter stressing ethnicity-specific disease. |  |
| *** Invasive, unpleasant, emotional or embarrassing procedures** | Reported in 10 SRs: (25, 30, 31, 39, 47, 53, 57, 83, 84, 90) | Belief about consequences (Reflective Motivation) or  Emotion (Automatic Motivation)  or  Environmental context and resources (Physical Opportunity) | Emphasising pain in information (−) | Emphasising risk in information. |  |
| *** Language or cultural barrier** | Reported in 9 SRs: (31, 39, 47, 57, 63, 71, 74, 75, 83) | Reinforcement (Automatic Motivation) or  Social Influences (Social Opportunity)  or  Environmental Context and resources (Physical Opportunity) |  | Letter stressing ethnicity-specific disease. | Educational programme on minority recruitment. |
| **Stigma associated with health condition** | Reported in 8 SRs: (35, 39, 56, 57, 69, 73, 82, 91) | Social influences (Social Opportunity) |  | SMS messages with quotes from existing participants. | Leaflet stressing PPI involvement. |
| **Uncertainty (particularly in relation to trials; its links to randomisation)** | Reported in 8 SRs: (23, 27, 38, 41, 70, 78, 81, 89) | Belief about consequences (Reflective Motivation) | Open trial design. | Total information disclosure.    Multimedia education about trials (DVD + booklet). | Patient preference trial design. |
| *** Lack of interest in research or lack of personal benefit** | Reported in 8 SRs: (25, 27, 44, 45, 57, 75, 83, 89) | Intentions (Reflective Motivation) |  | Positive framing of treatment effect. | Leaflet stressing PPI involvement. |
| *** Lack of knowledge about health condition or its treatment** | Reported in 6 SRs: (40, 63, 69, 70, 83, 84) | Knowledge (Psychological Capability) | Multimedia PIS. | Researcher reading out information (?).  Easy-to-read consent form.  Providing information by video.  Providing audio record of recruitment discussion.  Providing booklet on trial methods.  Total information disclosure.  Amended website wording plus infographic.    Multimedia plus paper PIS.  PPI designed PIS.  Researcher-optimised PIS.    Multimedia education about trials (DVD + booklet). | Brief patient information leaflet.  Optimising information through user testing or user feedback.  Multimedia PIS via QR code. |
| **Desire for choice** | Reported in 4 SRs: (23, 41, 51, 86) | Goals (Reflective Motivation) |  |  | Patient preference trial design. |
| *** Feeling of coercion, pressure or exploitation** | Reported in 4 SRs: (31, 70, 74, 75) | Social Influences (Social Opportunity)  or  Belief about consequences (Reflective Motivation) |  | Opt-out consent. |  |
| *** Personal experience of abuse or violence** | Reported in 1 SR: (76) | Emotion (Automatic Motivation) |  |  |  |
| *** Not feeling like a candidate for research** | Reported in 1 SR: (71) | Beliefs about Capabilities (Reflective Motivation)  or  Reinforcement (Automatic Motivation) |  | Opt-out consent. | Patient preference trial design. |
| **FACTORS REPORTED AS FACILITATORS OR BARRIERS** |  |  |  |  |  |
| **Influence of physician, family or friends, including support. (seen as important influence on decision).** | Reported in 28 SRs: (24, 32, 34, 38, 39, 42, 43, 46, 49, 50, 52, 55, 56, 59-61, 63, 69, 70, 74, 76, 77, 81, 83, 86, 87, 89, 92) | Belief about consequences (Reflective Motivation) or  Professional Role/ Identity (Reflective Motivation) |  | SMS messages with quotes from existing participants.  Decision aid about trials.  Handwritten, personalised letter. |  |
| **Information quality and quantity, and participant’s knowledge of the research** | Reported in 22 SRs: (25, 27, 31, 33, 38, 39, 42, 43, 47, 48, 52, 53, 55, 58, 63, 66, 69, 72, 75, 80, 83, 89) | Social influences (Social Opportunity) | Multimedia PIS. | Enclosing questionnaire on study method.  Researcher reading out information.  Easy-to-read consent form.  Shorter recruitment pack.    Providing information by phone.  Providing information by video.  Providing audio record of recruitment discussion.  Providing booklet on trial methods.  Total information disclosure.  Amended website wording plus infographic.    Multimedia plus paper PIS.    PPI designed PIS.  Researcher-optimised PIS. | Brief patient information leaflet.  Optimising information through user testing or user feedback.  Recruitment primer letter.  Multimedia PIS via QR code.  Educational programme on minority recruitment. |
| *** Sense of hope or sense of futility** | Reported in 10 SRs: (26, 28, 31, 32, 37, 54, 58, 64, 74, 88, 90) | Optimism (Reflective Motivation) |  | Positive framing of potential treatment benefits. |  |
| *** Attitude to healthcare or healthcare organisation or practitioners (positive or negative)** | Reported in 6 SRs: (39, 40, 64, 71, 89, 92) | Reinforcement (Automatic Motivation) or  Social Influences (Social Opportunity)  or  Professional Role/ Identity (Reflective Motivation) |  | SMS messages with quotes from existing participants.  Letter stressing ethnicity-specific disease. | Educational programme on minority recruitment. |
| *** Previous experience of research (positive or negative)** | Reported in 5 SRs: (49, 58, 71, 76, 83) | Reinforcement (Automatic Motivation) | Providing information in-person. | SMS messages with quotes from existing participants. | Leaflet stressing PPI involvement. |

(Factors newly identified in this review update have been marked in the table with an asterisk * in the first column)
